# Reversible Sterilization of Channel Catfish via Overexpression of Glutamic Acid Decarboxylase Gene

**DOI:** 10.3390/ani14131899

**Published:** 2024-06-27

**Authors:** Zhi Ye, Ahmed Elaswad, Baofeng Su, Ahmed Alsaqufi, Mei Shang, William S. Bugg, Guyu Qin, David Drescher, Hanbo Li, Zhenkui Qin, Ramjie Odin, Nonkonzo Makhubu, Nermeen Abass, Sheng Dong, Rex Dunham

**Affiliations:** 1 School of Fisheries, Aquaculture, and Aquatic Sciences, Auburn University, Auburn, AL 36849, USA; ahe0001@tigermail.auburn.edu (A.E.); bzs0014@auburn.edu (B.S.); aalsaqufi@kfu.edu.sa (A.A.); mzs0040@auburn.edu (M.S.); wsb0015@auburn.edu (W.S.B.); gzq0002@auburn.edu (G.Q.); ddrescher132@gmail.com (D.D.); hzl0026@auburn.edu (H.L.); qinzk@ouc.edu.cn (Z.Q.); ramjieodin@msumaguindanao.edu.ph (R.O.); nmakhubu@uniswa.sz (N.M.); n.y.abass@alexu.edu.eg (N.A.); szd0042@auburn.edu (S.D.); dunhara@auburn.edu (R.D.); 2 Key Laboratory of Tropical Aquatic Germplasm of Hainan Province, Sanya Oceanographic Institution, Ocean University of China, Sanya 572024, China; 3 MOE Key Laboratory of Marine Genetics and Breeding, College of Marine Life Sciences, Ocean University of China, Qingdao 266100, China; 4 Center of Excellence in Marine Biotechnology, Sultan Qaboos University, Muscat 123, Oman; 5 Department of Aquaculture and Animal Production, College of Agriculture and Food Sciences, King Faisal University, Al-Ahsa 31982, Saudi Arabia; 6 Department of Forest and Conservation Sciences, University of British Columbia, Vancouver, BC V6T 1Z4, Canada; 7 Division of Genetics, Department of Medicine, Brigham & Women’s Hospital, Harvard Medical School, Boston, MA 02115, USA; 8 Fisheries Department, Muckleshoot Indian Tribe, Auburn, WA 98092, USA; 9 College of Fisheries, Mindanao State University-Maguindanao, Datu Odin Sinsuat 9601, Philippines; 10 Department of Agricultural Botany, Faculty of Agriculture Saba-Basha, Alexandria University, Alexandria 21531, Egypt; 11 Department of Biological and Environmental Engineering, Cornell University, Ithaca, NY 14853, USA

**Keywords:** channel catfish, gamma-aminobutyric acid, transgenic fish, transgenic sterilization, hormone therapy

## Abstract

**Simple Summary:**

The present study was conducted to generate a reversibly sterile channel catfish that cannot reproduce without hormone therapy administered by man. This approach included transferring the glutamic acid decarboxylase (GAD) gene to channel catfish embryos at the one-cell stage to disrupt the migration of GnRH neurons and inhibit GnRH production. In natural spawning trials, transgenic fish exhibited generally lower spawning percentages than control fish of the same age. Two-thirds of the transgenic fish with repressed reproductive performance were successfully spawned after luteinizing hormone-releasing hormone analog (LHRHa) therapy. Transgenic fish exhibited lower serum gonadotropin-releasing hormone (GnRH) levels when compared with control fish. The overexpression of GAD repressed the reproductive performance of channel catfish, but the sterilization rate might be improved through selection in future generations.

**Abstract:**

The confinement of transgenic fish is essential to prevent their escape and reproduction in natural ecosystems. Reversible transgenic sterilization is a promising approach to control the reproduction of transgenic fish. Therefore, the present study was conducted to develop a reversibly sterile channel catfish (*Ictalurus punctatus*) via the transgenic overexpression of the goldfish (*Carassius auratus*) glutamic acid decarboxylase (GAD) gene driven by the common carp (*Cyprinus carpio*) β-actin promoter to disrupt normal gamma-aminobutyric acid (GABA) regulation. Three generations of GAD-transgenic fish were produced. All studied generations showed repressed reproductive performance; however, this was not always statistically significant. In F_1_, 5.4% of the transgenic fish showed a sexual maturity score ≥ 4 (maximum = 5) at five years of age, which was lower (*p* = 0.07) than that of the control group (16.8%). In the spawning experiments conducted on F_1_ transgenic fish at six and nine years of age, 45.5% and 20.0% of fish spawned naturally, representing lower values (*p* = 0.09 and 0.12, respectively) than the percentages in the sibling control fish of the same age (83.3% and 66.7%, respectively). Four of six pairs of the putative infertile six-year-old fish spawned successfully after luteinizing hormone-releasing hormone analog (LHRHa) therapy. Similar outcomes were noted in the three-year-old F_2_ fish, with a lower spawning percentage in transgenic fish (20.0%) than in the control (66.7%). In one-year-old F_2_-generation transgenic fish, the observed mean serum gonadotropin-releasing hormone (GnRH) levels were 9.23 ± 2.49 and 8.14 ± 2.21 ng/mL for the females and males, respectively. In the control fish, the mean levels of GnRH were 11.04 ± 4.06 and 9.03 ± 2.36 ng/mL for the females and males, respectively, which did not differ significantly from the control (*p* = 0.15 and 0.27 for females and males, respectively). There was no significant difference in the estradiol levels of the female transgenic and non-transgenic fish in the one- and four-year-old F_2_-generation fish. The four-year-old F_2_-generation male transgenic fish exhibited significantly (*p* < 0.05) lower levels of GnRH and testosterone than the control fish. In conclusion, while overexpressing GAD repressed the reproductive abilities of channel catfish, it did not completely sterilize transgenic fish. The sterilization rate might be improved through selection in future generations.

## 1. Introduction

**Figure 1:**
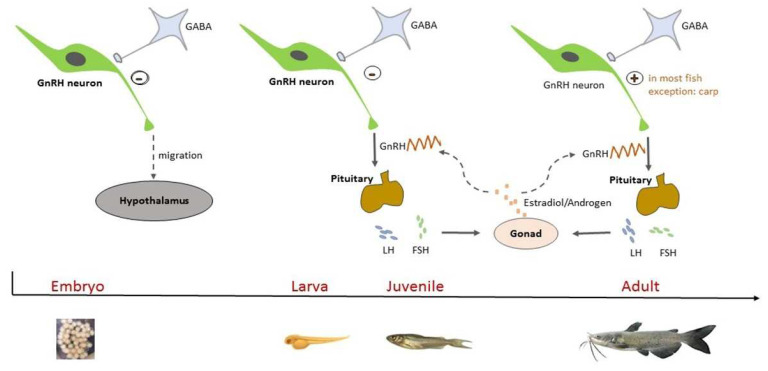
Involvement of γ-aminobutyric acid (GABA) in the regulation of reproductive activities in teleost fishes. GABA exerts an inhibitory effect on the migration of gonadotropin-releasing hormone (GnRH) neurons from the olfactory placode to the hypothalamus during embryogenesis. In most fish species studied so far, GABA showed an inhibitory and stimulatory effect on the production of GnRH before and after puberty, respectively. GABA also plays a role in the regulation of gonadotropin secretion and the positive and negative feedback of estrogen and androgen.

Gamma-aminobutyric acid (GABA) is one of the important factors involved in the guidance of gonadotropin-releasing hormone (GnRH) neuron migration [1,2]. It is formed from glutamic acid through a reaction catalyzed by glutamic acid decarboxylase (GAD), mainly in neurons in the central nervous system (CNS) [3]. GABA plays a role in nervous system development due to the close spatial relationship of GABA neurons and GnRH neurons [3,4] and the intimately associated expression patterns of GABA and GAD in the early developing zebrafish (*Danio rerio*) embryo before the formation of the synapse [5,6] (Figure 1). In the mouse, GABA works synergistically with stromal-derived growth factor (SDF-1) to slow or accelerate the migration of GnRH neurons by the activation of depolarizing or hyperpolarizing signaling pathways, respectively [7]. In another study, GABA was also proven to participate in the migration of GnRH neurons into the CNS [4]. Both in vivo and in vitro experiments of GABA_A_ receptor manipulation showed that GABA plays various roles in GnRH neuron migration at different development stages [8,9].

Transgenic and loss-of-function studies have further revealed the roles of GABA in GnRH neuron development. The overexpression of glutamic acid decarboxylase-67 (GAD67) in GnRH neurons disrupted the migration of GnRH neurons, resulting in fewer neurons reaching the hypothalamic-preoptic region and a lower GnRH hormone level during the first week of postnatal life in mice. The estrous cyclicity and reproductive capacity of transgenic females were disrupted, though the onset of puberty was unaffected [10]. The knockout of GAD67 genes in mice resulted in a 90% reduction in GABA in the developing brain [11], and the decreased levels of GABA accelerated the migration speed of GnRH1 neurons [7,12].

The migration of GnRH neurons from the olfactory placode to the hypothalamus during embryogenesis is critical for establishing and maintaining reproduction. When GnRH neuron migration is disrupted, the synthesis and release of GnRH will be affected, leading to abnormal reproductive function and possibly infertility [13]. Kallmann Syndrome, which is characterized by delayed or absent puberty and infertility, is a common disease caused by the incorrect migration of GnRH neurons in humans [14,15]. Female mice with the disrupted migration of GnRH neurons showed infertility or subfertility due to abnormal luteinizing hormone (LH) surges [16].

In addition to its role in GnRH neuron migration, GABA also affects reproduction by regulating LH release and puberty [17]. In most fish species studied so far, GABA showed an inhibitory effect on gonadotropin secretion at the larval stage, while an excitatory effect was reported in the adult fish [18]. In zebrafish, the expression of GnRH3 was inhibited by GABA in larval fish but stimulated in adult fish via the GABA_B_ receptor signaling pathway. The expression of LH and follicle-stimulating hormone (FSH) was also boosted by GABA in adult fish [19]. In vitro and in vivo studies conducted on adult female sea lampreys (*Petromyzon marinus*) have shown increased GnRH1 and GnRH3 hormone concentrations in the brain after the administration of GABA or its analog [20]. In adult dwarf gourami (*Trichogaster lalius*), the activation of the GABA_A_ receptor resulted in the excitation of the terminal nerve GnRH neurons [21]. In the Atlantic croaker (*Micropogonias undulatus*), the effect of GABA on LH secretion was mediated through GABA_A_ receptors, and the effect was stage dependent. GABA administration stimulated the expression of LH in croaker with regressed or pre-recrudescence-phase gonads, while it had no significant impact on fish in the early to middle recrudescence phase of the gonadal cycle and inhibited LH secretion in fish with fully recrudesced gonads [22]. Similarly, GABA injection increased serum gonadotropic hormone (GtH) levels in regressed or early-maturing goldfish (*Carassius auratus*) but did not affect late-maturing fish. The stimulatory effect of GABA on GtH was thought to be achieved indirectly through the increase in GnRH release [23]. There is also evidence that GABA stimulates LH secretion by blocking the inhibition of dopamine on LH release [24]. In rainbow trout (*Oncorhynchus mykiss*), GtH release in immature fish was not affected by GABA injection, while plasma LH in mature females was remarkably increased after GABA injection. In spermiating males, though the basal GtH release was not affected, GABA stimulated FSH secretion and potentiated GnRH-stimulated LH release when coadministered with GnRH analog (GnRHa) [25]. Nevertheless, the inhibitory effect of GABA on mature fish LH secretion was also observed in some fish species [26]. In the mature male common carp (*Cyprinus carpio*), it was suggested that GABA exerts an inhibitory effect on GnRH-stimulated LH release, probably through the GABA_B_ receptor [27] (Figure 1).

GABA is also involved in the positive and negative feedback of estrogen in both mammals and teleost fishes [28]. Typically, GnRH is under the negative feedback regulation of estrogen, but during the surge event before the ovulation, this feedback switches to be positive to initiate the surge of GnRH and GtHs. The change in GABA transmission frequency is regulated in an estradiol-dependent manner during the shift of the feedback action, which alters the GnRH neuron firing activity [3]. During negative feedback, the frequency of GABAergic postsynaptic currents is low, while it is increased along with enhanced amplitude at the surge onset [29]. Estradiol treatment in female goldfish not only abolished the stimulatory effect of GABA on GtH secretion but also reduced the GABA concentration in the telencephalon [23]. In immature rainbow trout, the influence of GABA on LH release changed from null to stimulatory 13 days after steroid implantation [25]. GnRH neurons do not express estrogen receptors (ER) [30], but ER was found in GABAergic neurons and dopaminergic neurons [31,32], which supported the important role of GABA in the feedback regulation of estrogen on GnRH [33] (Figure 1).

Two GAD genes (GAD65 and GAD67) that encode two GAD proteins with different molecular weights (65 kDa and 67 kDa), responsible for converting glutamic acid to GABA, have been isolated and characterized in multiple organisms [5,34,35,36,37]. GAD65 appears to be targeted to membranes and nerve endings [37], and the generated GABA is more likely used for vesicular release, while GAD67 is more widely distributed in cells and synthesizes cytoplasmic GABA [38]. In this study, we aimed to disrupt both the normal migration of GnRH neurons and the production of GnRH in channel catfish (*Ictalurus punctatus*) through the overexpression of the goldfish GAD65 gene driven by the common carp beta-actin promoter. Thus, the sexual maturation of channel catfish would be interrupted, which could then be restored by hormone therapy. The possibility of using this technology together with transgenic fish production could prevent the risk of potential environmental impacts from the transgenic fish since they would be unable to reproduce without hormone therapy administered by man.

## 2. Materials and Methods

### 2.1. Construction of the GAD65 Transgene Construct

**Figure 2:**
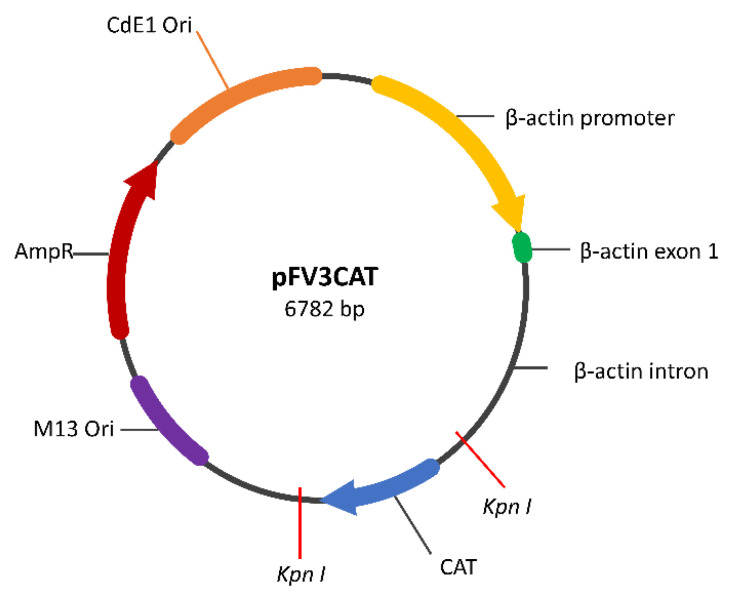
Map of the glutamic acid decarboxylase (GAD) transgene construct. A goldfish (*Carassius auratus*) GAD65 gene fragment cut with *BsrGI* restriction enzyme was inserted and ligated into the pFV3CAT vector by replacing the CAT fragment between the two *KpnI* restriction enzyme cutting sites (shown in red lines). The common carp (*Cyprinus carpio*) β-actin promoter in the upstream region drives the expression of GAD.

The transgene used in this study was constructed by AquaBounty Technologies (Maynard, MA, USA). Briefly, the goldfish GAD65 gene (GenBank accession number: AF045594.1) fragment, provided by Vance Trudeau, Ottawa University, was amplified with primers containing the *BsrGI* site and then inserted into pCR2.1 using the TOPO TA cloning kit (Invitrogen, Carlsbad, CA, USA). The recombinant vector was then digested with *BsrGI* restriction enzyme, and the GAD fragment was purified with a gel purification kit. The purified GAD fragment was fused into the *KpnI* site of the pFV3CAT vector (cut with *Acc65I* restriction enzyme, an isoschizomer of *KpnI*), which contains the common carp β-actin promoter in the upstream region (Figure 2).

### 2.2. Plasmid Preparation

The plasmids provided by AquaBounty were transformed into the One Shot^®^ Top 10 Chemically Competent *E. coli* cells (Invitrogen, Carlsbad, CA, USA) following the manufacturer’s instructions. Twenty-five microliters of the transformed *E. coli* culture were spread on an LB agar plate containing 100 μg/mL ampicillin [39]. A single colony was picked and inoculated into 500 mL LB containing 100 μg/mL ampicillin and cultured at 37 °C for 16 h. Plasmids were then extracted from the culture using the IsoPure Plasmid Maxi II Prep Kit (Denville, Holliston, MA, USA). The quality and quantity of the extracted plasmids were checked using a NanoDrop 2000 spectrophotometer (Thermo Scientific, Wilmington, DE, USA), and integrity was checked with electrophoresis. The concentration of the plasmid was adjusted to 50 ng/μL with TE buffer before electroporation.

### 2.3. Introduction of GAD65 Construct through Electroporation

Channel catfish broodstock were obtained from the ponds at the Fish Genetics Research Unit, School of Fisheries, Aquaculture and Aquatic Sciences, Auburn University, AL, USA. Females and males with well-developed secondary sex characteristics (well-rounded and distended abdomen for the females and muscular head and elongated urinogenital papillae for the males) were selected for artificial spawning. The general procedures for artificial spawning follow those described in [40,41]. Eggs were obtained by hand stripping. The males were sacrificed a few hours before the expected time of ovulation, and sperm were squeezed from the testes through a fine mesh and into 10 mL of 0.9% saline per gram of testes [40]. The sperm solution and stripped eggs were mixed, and pond water was added to activate fertilization. Twenty minutes after fertilization, 100 to 200 fertilized eggs were transferred to a 7 mL petri dish, and 3 mL of the plasmid solution was added to cover the eggs. After 10 min of incubation, the eggs were electroporated with a Baekon 2000 macromolecule transfer system (Baekon, Inc., Saratoga, CA, USA). Parameters were set at 6 kV, 2^7^ pulses, 0.8 s burst, 4 cycles, and 160 µs [42]. A control group electroporated with TE buffer only was also included.

### 2.4. Embryo Incubation

Electroporated embryos were incubated in 10 L tubs with 5.0 L Holtfreter’s solution (59 mM NaCl, 0.67 mM KCl, 2.4 mM NaHCO_3_, 0.76 mM CaCl_2_, 1.67 mM MgSO_4_) with 100 ppm doxycycline [43]. The embryos were gently agitated with compressed air delivered through an airstone. Holtfreter’s solution was changed every 12 h, and dead embryos were counted and removed before each solution change. Once the embryos hatched, the fry were transferred to fry baskets and temporally reared in a flow-through tank with pond water in the greenhouse.

### 2.5. Confirmation of Transgene Integration by PCR and Sequencing

Anal fin samples were collected from the fish for DNA extraction. Genomic DNA extraction was performed according to [44]. Briefly, samples were digested in cell lysis buffer (100 mM NaCl, 10 mM Tris, 25 mM EDTA, 0.5% SDS, 100 μg/mL proteinase K). After complete digestion, the protein was precipitated by adding 200 μL of protein precipitation solution (Qiagen, Redwood City, CA, USA). DNA was then precipitated with isopropanol, washed with 75% ethanol, dissolved in DNase-free ddH_2_O after air drying, and kept in the refrigerator overnight to allow for complete rehydration. The quality and quantity of extracted DNA were measured on a NanoDrop 2000 spectrophotometer, and integrity was checked by 1% agarose gel electrophoresis.

Primers targeting the boundary area of the β-actin promoter and the GAD65 gene were designed to confirm the transgene integration using the Primer Premier 6.0 software (PREMIER Biosoft International, San Francisco, CA, USA). The primers were checked for quality parameters such as GC content, primer dimers, hairpins, 3′ end stability, and melting temperature with Oligo Analyzer 3.1. They were also blasted against the channel catfish genome (IpCoco_1.2) from the National Center for Biotechnology Information (NCBI) database to ensure their specificity. Primers were first tested for specificity and efficiency by conducting PCR using the genomic DNA from non-transgenic channel catfish as the negative control template and the GAD plasmid DNA as the positive control template. The primer pair with no or minimal non-specific amplification in the negative control and the highest amplifying efficiency (with the brightest band shown by gel electrophoresis) was used for the following transgene screening. The same positive and negative controls were included in subsequent PCR reactions. The primer pair used was as follows: the forward primer sequence is 5′ TTGTCTGGCACATCTGAG 3′, and the reverse primer sequence is 5′ TACAATCACACCTGTCCAA 3′. The length of the PCR product was 274 bp. The PCR reaction was prepared in a 15 μL volume mix with the following components: 1.5 μL of 10 × buffer, 1.5 μL of 2.5 mM dNTP mix, 0.5 μL of 50 mM MgCl_2_, 0.75 μL of 10 μM of each forward and reverse primers, 0.4 U of *Taq* polymerase, and 200 ng of genomic DNA, brought up to 15 μL with ddH_2_O. The PCR program was as follows: initial denaturation for 5 min at 95 °C; followed by 39 cycles of denaturation at 95 °C for 30 s, annealing at 59 °C for 30 s, and extension at 72 °C for 30 s; and a final extension for 5 min at 72 °C. PCR results were checked by electrophoresis on 1% agarose gel.

In addition to PCR confirmation, the transgene sequence was further confirmed by Sanger sequencing. Briefly, the band at the correct size was cut off and purified with the QIAquick Gel Extraction Kit (Qiagen, Germantown, MD, USA) following the manufacturer’s instructions. The purified product was sent to the Auburn University Genomics and Sequencing Lab for sequencing. The sequence was confirmed by aligning it to the plasmid sequence.

### 2.6. Sexual Maturation, Fertility Evaluation, and Hormone Therapy of F_1_ and F_2_ Fish

The F_1_ generation was produced from positive P_1_ fish using artificial spawning as described above. Fish were harvested during the spawning season at five years of age to assess sexual maturity. Each fish was given a score from 1 to 5 according to the secondary sex characteristics. Score 5 was the highest and was characterized by a well-rounded and distended abdomen, open red genitalia for the females, and a very muscular head and elongated urinogenital papillae for the males; score 4 was characterized by rounded and distended abdomen, open red genitalia for the females, and a muscular head and elongated urinogenital papillae for the males; score 3 described a fish with a rounded abdomen, open, light red genitalia for the females, and a medium muscular head and elongated urinogenital papillae for the males; score 2 meant a fish with a slightly rounded abdomen, not swollen genitalia for the females, and a normal head and slightly elongated urinogenital papillae for the males; and score 1 was the lowest and was assigned to fish with a flat abdomen, not swollen, pale genitalia for the females, and a normal head and small, soft urinogenital papillae for the males.

Six- and nine-year-old F_1_ GAD-transgenic fish and control fish were paired with the following combination: GAD ♀ × control ♂; control ♀ × GAD ♂; and control ♀ × control ♂. Each pair was placed in an individual 120 L flow-through aquarium wrapped with black plastic film and given a photoperiod of 12 h/12 h. The water was aerated with compressed air delivered through an airstone to maintain the dissolved oxygen level above 7 ppm. Water quality was monitored and kept within the safe range for catfish (nitrite = 0 ppm, ammonia = 0 ppm, pH = 6.8~8). The fish were allowed to spawn naturally for 14 days. The fertility of fish was determined by the successful production of fertilized eggs. Fish that did not spawn for 14 days during a natural spawning trial were classified as putatively infertile and were provided with hormone therapy, that is, receiving an LHRHa implant at the dosage of 90 μg per kilogram fish weight to induce spawning. The implanted fish were returned to the aquarium, and another seven-day spawning trial was conducted.

The embryos (F_2_) from the GAD-transgenic fish, which spawned after hormone therapy, were incubated in a flow-through hatching. The hatched fry were cultured in flow-through tanks with similar pond water to that of the P_1_ fish. The fry were fed with powdered 50% protein starter feed at approximately 3 days post-hatching (dph) and pelleted 36 to 48% protein feed as the fingerlings grew. Once they reached 10 g, the fish were stocked into a 404.7 square meter pond with a 1 m depth of water at a density of approximately 0.7 fish per cubic meter of water until maturation. Fish were fed ad-libitum with commercial floating catfish feed containing 32 to 36% protein once every day. Feeding rates were reduced when the temperature was lower. F_2_ GAD-transgenic fish received the same spawning trial as described above for fertility evaluation at 3 years of age.

### 2.7. Measuring of Serum GnRH, Estradiol, and Testosterone Levels by ELISA

Blood was collected from the F_2_ GAD-transgenic fish in June at one and four years of age. Fish were anesthetized with 100 ppm buffered tricaine methane sulfonate (MS 222), and 0.5 and 1.0 mL blood samples were collected from the caudal vein of the one- and four-year-old fish, respectively, using a sterile syringe. The four-year-old fish were placed back in the tank with clean water to recover, while the one-year-old fish were euthanized with 400 ppm buffered MS222 after sampling. Blood samples were stored at 4 °C overnight to clot and then centrifuged at 1000× *g* for 15 min to isolate the serum. The serum was aliquoted and stocked in a −80 °C freezer until the hormone measurement. Hormone levels in the serum were measured using ELISA kits (GnRH and estradiol kits were from CUSABIO Biotech Corp., Ltd., Baltimore, MD, USA; the testosterone kit was from Cloud-Clone Corp., Houston, TX, USA) according to the manufacturer’s instructions. Serum hormone levels were measured at one and four years of age. GnRH levels were measured in both sexes, while estradiol and testosterone levels were measured in females and males, respectively.

### 2.8. Statistical Analysis

All the data analyses were conducted using the SAS 9.2 program. Survival analysis of the P_1_ embryos was performed using GraphPad Prism 6. The log-rank test was used to compare the P_1_ embryo survival curves. Fisher’s exact test was used to analyze the sexual maturation evaluation data and the spawning percentage data. The Chi-square test was used to test the transgenic ratio in the F_2_ generation at the 50% expectation level. ANOVA (one-way, two-way) was used to analyze the body weight and hormone levels. The significance for all tests was set at *p* < 0.1. Data are presented as the mean ± standard deviation (SD).

## 3. Results

### 3.1. Mortality of the P_1_ Embryos

Both the control and GAD construct-exposed channel catfish embryos exhibited a rapid decline in survival during the 1.5 to 3.5 days post-fertilization (dpf) period. There was no significant difference (*p* = 0.94) in the survival curves of the GAD construct-exposed and control embryos.

### 3.2. Identification of Transgenic F_2_ Progenies and Growth Evaluation

**Figure 3:**
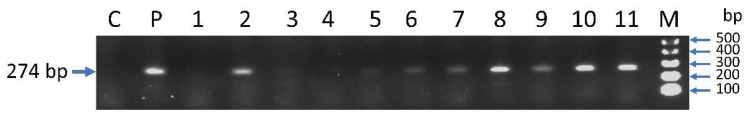
Identification of glutamic acid decarboxylase (GAD)-transgenic channel catfish (*Ictalurus punctatus*) with primers targeting the β-actin promoter GAD gene area. The GAD65 gene was obtained from goldfish (*Carassius auratus*) and was driven by the common carp (*Cyprinus carpio*) β-actin promoter. Forward and reverse primers were designed from the β-actin promoter and GAD65 gene region, respectively. The size of the target amplicon was 274 bp. Numbers 1–11 represent the tested samples; C: non-transgenic control channel catfish; P: positive control using the GAD plasmid as the template for PCR; M: DNA ladder.

In F_2_, 73 out of the 150 (48.7%) tested fish were transgenic, as identified by PCR (Figure 3) and confirmed by DNA sequencing. The transgenic ratio was consistent with the theoretical ratio of 1:1 when one of the parents is a heterozygous transgenic fish and the other is non-transgenic (*p* = 0.74). No significant difference (*p* = 0.64) was observed in the body weight of the one-year-old full-sib transgenic (10.96 ± 4.33 g) and non-transgenic (11.22 ± 2.87 g) F_2_ progenies.

### 3.3. Gravidity, Fertility Assessment, and Hormone Therapy of the F_1_ and F_2_ Generations

At five years of age, none of the 37 F_1_ GAD-transgenic females (14) or males (23) exhibited a sex maturity score of 5, and only one female and one male received a score of 4 (Table 1). When the sexes were combined, the percentage of GAD transgenics at a reproductive readiness of 4 or 5 was less (*p* = 0.07) than that of non-transgenic controls.

In a spawning trial conducted using six-year-old F_1_ fish, 83.3% (10 out of 12) of the control channel catfish pairs spawned naturally without LHRHa implants, while only 45.5% (5 out of 11) of the GAD fish pairs spawned naturally, which was significantly lower (*p* = 0.09) than the controls. Four of the six pairs of GAD fish that did not spawn naturally did so after induction with LHRHa (Table 2). In the spawning trial conducted using the nine-year-old fish, the GAD-transgenic fish showed a lower percentage of spawning than the controls (20% and 66.7%, respectively), but this difference was not statistically significant (*p* = 0.12; Table 2).

For the F_2_ generation, among the three-year-old GAD-transgenic fish, only 20% (3 of 15) spawned naturally, which was lower than in the control group (66.7%) but not significant (*p* = 0.12). None of the five pairs of infertile GAD fish, which were still in good spawning condition after the natural spawning trial and received LHRHa therapy, had fertility restored (Table 2). 

### 3.4. Hormone Levels in the F_2_ Fish

The difference in GnRH levels between the GAD and control fish was not significant within each sex (*p* = 0.15 and 0.27 for females and males, respectively). No significant difference in GnRH levels between female and male fish was observed within each genotype group (*p* > 0.05). No genotype × sex interaction was observed (*p* = 0.77). The estradiol level in the transgenic female fish serum was not different (*p* = 0.78) from that of the non-transgenic female fish (Table 3).

For the four-year-old fish, the GAD-transgenic males exhibited significantly (*p* = 0.012) lower GnRH levels than the control male fish, but the difference was not significant (*p* > 0.1) between the transgenic and non-transgenic female fish. GnRH levels in different sexes were also significantly different (*p* = 0.002), with females having higher GnRH levels than males. When split by genotype, only the GAD females had significantly higher (*p* = 0.0028) GnRH levels than the GAD males. Serum GnRH levels were not significantly different in control males and females (*p* = 0.39) (Table 4). There was no difference (*p* = 0.9069) observed between the GAD-transgenic and non-transgenic control female fish for estradiol levels. The GAD-transgenic male fish exhibited significantly (*p* = 0.0018) lower testosterone levels than the non-transgenic males (Table 4).

## 4. Discussion

Three generations of GAD-transgenic fish were produced, and reproductive performance was repressed in the studied generations, as revealed by sexual maturation evaluation and spawning percentages. The reproductive performance of the putative infertile GAD-transgenic fish was not always restored by hormone therapy. GnRH levels in the sera of one-year-old and four-year-old F_2_-generation fish showed the same trend that no significant difference existed between the female transgenic and non-transgenic fish. The lower GnRH levels in the four-year-old male fish implied a different regulation mode of GABA on GnRH in adult males than in females, as transgenic and control females were not different.

Decreased survival due to transgene introduction would be a negative pleiotropic effect, and any negative pleiotropic effect could negate the benefit of transgenic sterilization. The rapid decline in survival in both the control and GAD construct-exposed channel catfish embryos during the 1.5 to 3.5 days post-fertilization (dpf) period is consistent with the results observed previously in a similar Bax gene overexpression experiment conducted on the same species [45]. The introduction of transgenic constructs into one-cell embryos may affect survival. In channel catfish, several transgenic constructs significantly reduced the embryo hatch rate compared with that of control embryos electroporated with TE buffer [46]. In the current study, there was no significant difference in the survival curves of the GAD construct-exposed and control embryos, indicating that the introduction of GAD did not increase the mortality of channel catfish embryos.

Growth rate is another economically important trait for which adverse pleiotropic effects from transgene insertion would be a concern. As one of the major inhibitory neurotransmitters in the CNS, GABA is involved in multiple biological functions [18,47,48,49,50,51]. A study on humans showed that the oral administration of GABA could elevate serum growth hormone concentration [52]. Another study indicated growth enhancement and upregulation of growth-related gene expression in zebrafish larvae exposed to different concentrations of GABA at three days post-fertilization [53]. In the current study, overexpressing the GAD gene did not influence growth, as indicated by the similar growth rates in one-year-old F_2_-generation full-sib GAD-transgenic and non-transgenic fish. This may be due to species differences and the effect of GABA concentrations. In the zebrafish study, GABA enhanced larval growth in a concentration-dependent manner, with the best improvement resulting from 25 and 50 mM concentrations [53]. These concentrations are very high compared with the endogenous GABA concentrations in the brain tissue (1–5 µmol) [54]. The transgene insertion was also reported to affect more than one trait positively or negatively. Therefore, future studies should evaluate other aspects of pleiotropic effects of GABA, including disease or stress resistance, survival, and seinability, as GABA was also demonstrated to be effective as a relaxant and could enhance immunity under stress conditions in humans and chickens [55,56,57].

The ultimate objective of this study was to affect fertility and restore fertility. The current results revealed a significantly lower sex maturity score in GAD-transgenic fish at five years of age, indicating that the sexual maturation of the GAD-transgenic fish was inhibited to some extent, and their reproductive performance was disrupted. Three years is the age threshold of reproductive capability for most channel catfish strains [58], although early sexual maturity at two years of age is possible. The spawning trials conducted using six-year-old F_1_ fish indicated a significantly lower natural spawning percentage in GAD fish than in control fish. Following LHRHa injection, the natural spawning was restored in two-thirds of the GAD fish that did not spawn naturally. These results showed that the overexpression of GAD could lead to impaired reproduction in channel catfish, and fertility could be restored by hormone therapy. However, sexual maturation was not always restored, indicating that hormone therapy to restore fertility needs improvement by testing different dosages, different times or frequencies of hormone injection, and possibly hormone combinations. Alternatively, the experimental design delayed the hormone application for a significant time to prove sterility. This delay may, in some cases, have led to the fish being too far into the spawning season to restore fertility.

In regard to fertility, the roles of GABA in teleost fish reproduction discovered so far consist of guiding GnRH neuron migration during embryogenesis, regulating GnRH secretion in different life stages, and mediating LH secretion and estradiol feedback regulation. The overexpression of the GAD gene should lead to an increase in GABA, which in turn would interfere with the normal regulation of GABA in these reproduction-related biological processes, thereby disrupting normal reproductive activities. However, studies have shown that the interruption of GnRH neuron migration or the production of GnRH may not be sufficient to cause major problems in reproduction, in contrast to the reduced reproduction that we observed in GAD-transgenic channel catfish. In GAD67-transgenic mice, the overexpression of GAD slowed the migration of GnRH neurons and resulted in increased positional diversity of these neurons but did not block the neurons from migrating into the brain. The reproduction of the mutant mice was not totally interdicted but was affected to a certain extent, as the mutant mice exhibited normal puberty initiation but inordinate estrous cyclicity and reduced reproductive capacity [10]. Another study in mice demonstrated that a small portion (12%) of the GnRH neuron population was sufficient for puberty onset, and the dysfunction of the majority of GnRH neurons is required to disrupt fertility [16]. Similarly, mice with the GABA_A_ receptor knocked down in GnRH neurons exhibited normal puberty onset, cyclicity, and fertility, but the negative feedback of estrogen on LH was affected, with the increment of LH in the GABA_A_ KO mice two weeks after ovariectomy being almost double of that in the control mice [59].

Again in contrast to our results, the bi-allelic knockout of the zebrafish GnRH3 gene did not cause major changes in ontogeny and reproduction, and the higher levels of gonadotropin gene mRNA observed in the early developing mutant fish were also adjusted to normal levels in the adults [60]. The partial ablation of GnRH3 neurons in zebrafish caused a reduction in fecundity, but oocyte development was normal, and the fish were fertile, while fish with GnRH3 neurons completely ablated were infertile [61]. In our study, although not always statistically significant, GAD-transgenic fish showed reduced reproductive capacity consistently in the F_1_ and F_2_ generations as compared to the non-transgenic controls. The less than 100% disruption of reproduction by GAD overexpression in this study might be due to the incomplete disruption of GnRH neuron migration so that some of the neurons migrated to their destination, which could be sufficient to partially regulate the proper development of the reproductive system. If controlled properly, the incomplete disruption of GnRH neurons could be beneficial, as the ultimate goal was to make the sterilization reversible so that the reproduction of the transgenic fish could be restored when the production of the next generation is desired. In some cases, the restoration of fertility was unsuccessful in the current study; therefore, the optimization of the hormone therapy strategy is needed to increase the success of fertility restoration, as the efficiency of hormone therapy could be affected by the dosage, timing, and frequency of hormone administration [62,63]. Additionally, our gene insertion was random. The integration site may have affected the extent of reproductive repression. If this is the case, selection might allow for a further decrease in reproduction or complete sterility.

Theoretically, reduced reproduction should be related to changes in GnRH production. GABA exhibited an inhibitory effect on the secretion of GnRH in immature fish and a stimulatory effect on mature fish for most fish species [18]. In this study, observed GnRH levels in the one-year-old transgenic fish sera were lower than in the non-transgenic fish for both the females and males, but the difference was not statistically significant. The difference was significant when the sexes were pooled. In addition to the inhibitory effect of GABA on GnRH secretion, GAD-transgenic fish should have fewer functional GnRH neurons and thereby less GnRH secreted if the migration of GnRH neurons is successfully disrupted by the overexpression of GAD, assuming other compensatory pathways do not exist in these fish. In GAD-transgenic mice, GnRH levels in the hypothalamus were lower than those in the control mice, but they were quickly adjusted back to normal values by the second week after birth. Accordingly, GtH levels in the serum exhibited the same change and were returned to similar levels to those in the control mice by four weeks of age [10]. Similar compensatory mechanisms might also exist in the GAD-transgenic fish to adjust their hormone synthesis and secretion so that they could address the problems of insufficient GnRH neurons. In the four-year-old fish, there was no significant difference in the GnRH and estradiol levels between the transgenic and non-transgenic female fish. However, GnRH levels in the GAD-transgenic males were lower than those in the non-transgenic males. Correlation analysis showed that the GnRH level was positively correlated with the level of estradiol and testosterone in the four-year-old female (*R* = 0.96, *p* = 0.0001) and male (*R* = 0.73, *p* = 0.17) fish, respectively. However, there was no correlation between the levels of GnRH and estradiol in the one-year-old female fish (*p* = 0.13). This discrepancy in the correlations between the GnRH and sex steroid levels in the one-year-old and four-year-old fish is likely due to the difference in life stages, as the feedback regulation of sex steroid in fish is age, season, and reproductive stage dependent [64,65,66,67].

## 5. Conclusions

The overexpression of GAD showed some potential to repress the reproductive performance of fish. A small percentage of GAD-transgenic channel catfish were fertile. The overexpression of GAD may not be 100% effective, as compensatory mechanisms may exist, or there may be allelic differences in the transgene insertion, leading to the fertility of this small percentage of GAD individuals. These potential factors should be evaluated in future studies. Additionally, selection for individuals that exhibit the appropriate sterile phenotype may lead to 100% effectiveness of this approach. The hormone therapy was not always effective, and this is not surprising since GnRH is not responsible for the last step in gamete maturation and release. A more complex regime of GnRH administration may be needed to lead to a more consistent restoration of fertility and spawning.

## Figures and Tables

**Table 1:** Percentages of F_1_-generation glutamic acid decarboxylase (GAD)-transgenic and non-transgenic channel catfish (*Ictalurus punctatus*) with a reproductive score of 5 or 4 at five years of age. The GAD65 gene from goldfish (*Carassius auratus*) was driven by the common carp (*Cyprinus carpio*) β-actin promoter. When data for both sexes were combined, GAD-transgenic fish showed lower percentages (*p* = 0.07, Fisher’s exact test) of fish with a reproductive score of 4 or 5 compared to the non-transgenic controls. Percentages followed by different superscript letters are significantly different (*p* < 0.1).

Sex	Genotype	Total Number	Reproductive Score *		
			5	4	4 & 5
Female	Control	227	22 (9.7%)	25 (11%)	47 (20.7%)
	GAD	14	0	1 (7.1%)	1 (7.1%)
Male	Control	232	10 (4.3%)	20 (8.6%)	30 (12.5%)
	GAD	23	0	1 (4.3%)	1 (4.3%)
Both sexes	Control	459	32 (7.0%)	45 (9.8%)	77 (16.8%) ^a^
	GAD	37	0	2(5.4%)	2 (5.4%) ^b^

* Score 5 was characterized by a well-rounded and distended abdomen, open red genitalia for the females, and a very muscular head and elongated urinogenital papillae for the males. Score 4 indicated a rounded and distended abdomen, open red genitalia for the females, and a muscular head and elongated urinogenital papillae for the males. Any score of 3 or lower represents poor or no spawning readiness.

**Table 2:** Spawning percentages of glutamic acid decarboxylase (GAD)-transgenic channel catfish (*Ictalurus punctatus*) at six and nine years of age (F_1_) and at three years of age (F_2_) under natural aquarium spawning conditions and after hormone therapy (90 μg LHRHa/kg body weight). The GAD65 gene from goldfish (*Carassius auratus*) was driven by the common carp (*Cyprinus carpio*) β-actin promoter. Spawning trials were conducted using six-year-old F_1_ GAD-transgenic fish and three years later using nine-year-old F_1_ and three-year-old F_2_ fish. GAD-transgenic fish were individually paired with non-transgenic control fish from the opposite sex and were allowed to spawn naturally for 14 days. If not spawned, hormone therapy was applied to the fish that were still in good spawning condition, and fish were allowed to spawn for an additional seven days. In all the spawning trials, GAD-transgenic fish exhibited lower percentages of natural spawning than the controls; however, this was only significant in the six-year-old F_1_ fish (*p* = 0.09, Fisher’s exact test) compared with *p* = 0.12 for the nine-year-old F_1_ and the F_2_ fish. Combining all generations revealed a significantly lower spawning percentage in GAD-transgenic fish (27.8%, *p* = 0.0005) than in control fish (75%). N is the number of fish pairs for the spawning trials.

Trial	Genotype	Without Implant *			With Implant		
		N	Pairs Spawned	%	N	Pairs Spawned	%
Six-year-old F_1_	Control	12	10	83.3	-	-	-
	GAD	11	5	45.5	6	4	66.7
Nine-year-old F_1_	Control	6	4	66.7			
	GAD	10	2	20.0	3	0	0.0
Three-year-old F_2_	Control	6	4	66.7	-	-	-
	GAD	15	3	20.0	5	0	0.0
Combined generations	Control	24	18	75.0 ^a^	-	-	-
	GAD	36	10	27.8 ^b^	14	4	28.6

* Means in the same column followed by different superscript letters (^a,b^) are significant (*p* < 0.05, Fisher’s exact test).

**Table 3:** Means ± standard deviation of serum gonadotrophin-releasing hormone (GnRH) and estradiol levels of the one-year-old F_2_ glutamic acid decarboxylase (GAD)-transgenic and full-sib non-transgenic channel catfish (*Ictalurus punctatus*). The GAD65 gene from goldfish (*Carassius auratus*) was driven by the common carp (*Cyprinus carpio*) β-actin promoter.

Genotype	Hormone *			
	GnRH (ng/mL)		Estradiol (pg/mL)	Testosterone (ng/mL)
	Female	Male	Female	Male
GAD	9.23 ± 2.49 (n = 24)	8.14 ± 2.21 (n = 20)	501.00 ± 69.96 (n = 24)	ND
Control	11.04 ± 4.06 (n = 10)	9.03 ± 2.36 (n = 11)	520.48 ± 148.31 (n = 10)	ND

* Blood samples were collected in June when the temperature was approximately 28 °C. Means of sexes/genotypes were compared using ANOVA (*p* < 0.05). ND, not detectable.

**Table 4:** Means ± standard deviation of serum gonadotrophin-releasing hormone (GnRH), estradiol, and testosterone levels in the four-year-old F_2_ glutamic acid decarboxylase (GAD)-transgenic and non-transgenic channel catfish (*Ictalurus punctatus*). The GAD65 gene from goldfish (*Carassius auratus*) was driven by the common carp (*Cyprinus carpio*) β-actin promoter.

Genotype	Hormone *			
	GnRH (ng/mL)		Estradiol (pg/mL)	Testosterone (pg/mL)
	Female	Male	Female	Male
GAD	3.05 ± 1.22 ^a^ (n = 6)	1.02 ± 0.31 ^b^ (n = 6)	262.17 ± 82.25 (n = 6)	288.84 ± 62.62 ^A^ (n = 6)
Control	3.07 ± 0.66 ^a^ (n = 6)	2.34 ± 1.22 ^a^ (n = 4)	254.14 ± 71.73 (n = 6)	699.12 ± 211.90 ^B^ (n = 4)

* Blood samples were collected in June when the temperature was approximately 28 °C. Means of genotypes were compared using ANOVA. There was no genotype × sex interaction (*p* > 0.05) in gonadotropin-releasing hormone (GnRH) levels. Means in the same row (for GnRH) or column (for GnRH, estradiol, or testosterone) followed by different superscript letters (lowercase for GnRH and uppercase letters for testosterone) were significantly different (*p* < 0.05).

## Data Availability

All the data generated from the current study are included in the manuscript.
